# Pharmacokinetics of Meropenem in People with Cystic Fibrosis—A Proof of Concept Clinical Trial

**DOI:** 10.3390/antibiotics10030292

**Published:** 2021-03-11

**Authors:** Jan C. Kamp, Jan Fuge, Felix C. Ringshausen, Denis Grote-Koska, Korbinian Brand, Lukas Graalmann, Ralf-Peter Vonberg, Tobias Welte, Jessica Rademacher

**Affiliations:** 1 Department of Respiratory Medicine, Hannover Medical School, 30625 Hannover, Germany; Kamp.jan-christopher@mh-hannover.de (J.C.K.); Fuge.jan@mh-hannover.de (J.F.); Ringshausen.felix@mh-hannover.de (F.C.R.); graalmann.lukas@mh-hannover.de (L.G.); welte.tobias@mh-hannover.de (T.W.); 2 Biomedical Research in Endstage and Obstructive Lung Disease Hannover (BREATH), German Center for Lung Research (DZL), 30625 Hannover, Germany; 3 Institute of Clinical Chemistry, Hannover Medical School, 30625 Hannover, Germany; Grote-Koska.Denis@mh-hannover.de (D.G.-K.); brand.korbinian@mh-hannover.de (K.B.); 4 Institute for Medical Microbiology and Hospital Epidemiology, Hannover Medical School, 30625 Hannover, Germany; Vonberg.ralf@mh-hannover.de

**Keywords:** cystic fibrosis, carbapenem, pharmacokinetics, pulmonary exacerbation, antimicrobial stewardship

## Abstract

Anti-infective treatment of pulmonary exacerbations is a major issue in people with cystic fibrosis (CF). Individualized dosing strategies and adaptation of infusion times are important concepts to optimize anti-infective therapy. In this prospective non-randomized controlled open-label trial, we compared pharmacokinetics of meropenem in 12 people with CF experiencing a pulmonary exacerbation, of whom six received parenteral meropenem 2 g tid as short infusion over 30 min and six extended infusion over 120 min. We measured blood concentrations of meropenem at five predetermined time points over 240 min and calculated differences in the percentages of the time above the minimal inhibitory concentration (fT > MIC) for meropenem concentrations >16 and >32 mg/L, respectively. Mean percentages of fT > 16 and fT > 32 mg/L were higher in the extended compared to the short infusion group (83 and 56% vs. 59% and 34%), with a statistically significant prolongation of the fT > 32 mg/L (mean 134 vs. 82 min; *p* = 0.037). Our results demonstrate that, in people with CF, longer fT > MIC can be achieved with a simple modification of meropenem dosing. Further studies are needed to clarify if this may translate into improved microbiological and clinical outcomes, in particular in adults with difficult-to-treat chronic infection by carbapenem-resistant *Pseudomonas aeruginosa*.

## 1. Introduction

Pulmonary exacerbations frequently occur in people with cystic fibrosis (CF). The most important pathogen chronically infecting the airways of people with CF is *Pseudomonas aeruginosa* (PA) [1], which may cause severe pulmonary exacerbation requiring high-dose intravenous (IV) treatment. Meropenem is an important antibiotic agent for the treatment of exacerbations in CF, which in general has been effective and well tolerated according to previous studies [2,3,4]. The efficacy of meropenem in treating multidrug-resistant (MDR) PA is of particular importance [5]. In people with CF, augmented doses of meropenem (2 g IV tid) have been observed to exceed the susceptibility breakpoint (4 mg/L) around 50% for the dosing interval, which was longer compared to standard doses (1 g IV tid) [6] due to an increased clearance of beta-lactams in this patient population [7,8]. Antibiotic treatment duration of at least two weeks has been shown to obtain satisfying pulmonary function benefit [9], and often, it is started as in-patient therapy and continued as home IV antibiotic therapy (HIVAT) [10]. Although 3-h extended infusion of meropenem has been shown to be beneficial in people with CF due to enduring target plasma concentrations in a small case-series [3], there still is a lack of data about its clinical relevance and feasibility for HIVAT. Moreover, the therapeutic range of meropenem is not consistently defined in the literature. In a multicenter study, the minimal inhibitory concentration (MIC50/MIC90) recovered from 414 PA isolates cultured from respiratory samples of people with CF was 0.25 respectively 16 mg/L [11]. Applying both European Committee on Antimicrobial Susceptibility Testing (EUCAST) and Clinical and Laboratory Standards Institute (CLSI) breakpoints (defining resistance at a MIC > 8 mg/L), 18% of the strains were resistant to meropenem [11]. In patients with MICs > 8 mg/L, it is advisable to treat with meropenem in the case that higher plasma levels (4–6 times the MIC) can be reached around 50% of infusion time without relevant side effects. While spontaneous resistance selection in PA may be suppressed with optimized meropenem exposure, no toxic threshold has been defined yet, and high doses of meropenem may cause liver injury and neurotoxicity. In the present study, we compared the pharmacokinetics of meropenem in both short infusion (30 min) and extended infusion (120 min) in 12 consecutive people with CF admitted to our hospital with pulmonary exacerbation. We hypothesized that extended is superior to short infusion in achieving enduring target plasma meropenem levels in people with CF to overcome the endemic strains with lower susceptibility to antibiotics. Our aim is the improvement of IV antibiotic treatment, feasible as HIVAT, by small changes of the dosing strategy.

## 2. Methods

In this investigator-initiated prospective non-randomized controlled open-label trial, we enrolled 12 adults with CF and normal renal function (glomerular filtration rate ≥60 mL/min), who were admitted to our hospital due to a pulmonary exacerbation. The first 6 patients received meropenem in a dose of 2 g over 30 min tid, while the second 6 patients received the same dose regimen over 120 min. Patients were not stratified for clinical characteristics or laboratory findings. On the second day of antibiotic treatment, we measured blood concentrations of meropenem at predetermined time points (trough level as well as concentrations 30, 60, 120, and 240 min after starting the infusion). Measurements were conducted via a certified high performance liquid chromatography (HPLC) method (details in the supplement).

We compared the time of the free drug concentrations above the minimal inhibitory concentration (fT > MIC) in both short and extended infusion regimens. The fT > MIC has repeatedly been shown to be the pharmacodynamic variable most closely linked to bactericidal activity [12]. Because of the above-mentioned diversity in antibiotic resistance properties of different PA strains, we determined the fT > MIC for meropenem concentrations >16 and >32 mg/L. Additionally, we assessed peak meropenem concentrations and compared these findings between the two infusion regimens.

All patients were consecutively recruited between October 2019 and September 2020. Institutional Review Board approval (No. 8702_BO_K_2019) was given to our study according to German regulations and all patients provided written and informed consent. All patients fulfilled the following inclusion criteria: (i) a confirmed diagnosis of CF according to European standards of care [13]; (ii) age ≥18 years, (iii) normal renal function at enrolment, assessed by chronic kidney disease epidemiology collaboration (CKD–EPI)-formula estimated glomerular filtration rate ≥60 mL/min, and (iv) in-patient initiation of antibiotic treatment with meropenem for pulmonary exacerbation. There were no further exclusion criteria. The medical indication for meropenem treatment was based on the discretion of the treating CF physician in accordance with current national treatment guidelines [14]. The manuscript was written in accordance with the STROBE reporting checklist for cohort studies.

### Statistical Analysis

We used SPSS Statistics 27.0 (IBM Corp, Armonk, NY, USA) and Stata 13.0 (State Corp LP, College Station, TX, USA) statistical software to analyze the data. Continuous variables are shown as median and interquartile range or mean and standard deviation, as appropriate. Categorical variables are shown as numbers and percentage (%). For group comparisons, Fisher’s exact test, Chi-squared test, or Mann–Whitney U-test were used, as appropriate. Normal distribution of continuous variables was checked by Kolmogorov–Smirnov test. Linear equations were used to calculate the assumed time point of reaching therapeutic plasma levels. All reported *p*-values are two sided. *p*-values < 0.05 were considered statistically significant.

## 3. Results

A total of 15 consecutive adults with CF experiencing a pulmonary exacerbation were screened for eligibility of whom 12 were included in this analysis and three were excluded because of impaired renal function. A total of 12 consecutive adults were included in this analysis, of whom six received meropenem over 30 and 120 min, each. Baseline characteristics were obtained prior to meropenem treatment initiation and were comparable between groups (Table 1).

The mean percentages of fT > 16 and fT > 32 were higher among patients who received meropenem over 120 min (extended infusion group) compared to 30 min (short infusion group; 83% and 56% of 240 min vs. 59% and 34%, respectively). However, a statistically significant prolongation could only be shown for the time above 32 mg/L (*p* = 0.037; Table 2).

**Figure 1:**
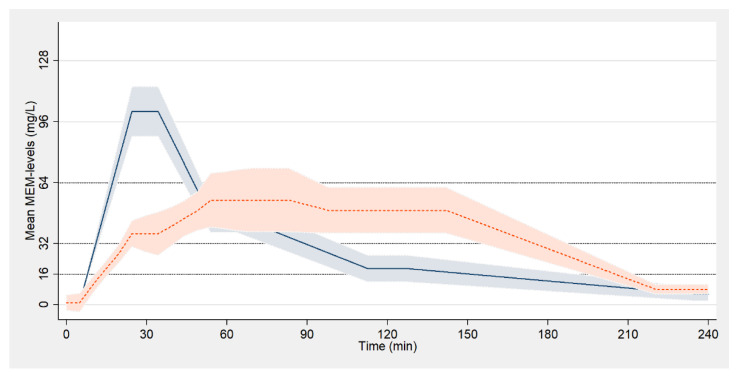
Pharmacokinetics of meropenem (color printing). Pharmacokinetics are presented as polynomial smoothed line with 95% confidence interval. Patients who received meropenem over 30/120 min are blue-/orange-colored, respectively.

The mean peak meropenem concentration was significantly higher in patients who received meropenem over 30 min (*p* = 0.005; Table 2). Pharmacokinetics of meropenem in both groups are visualized in Figure 1.

The respective raw data can be found in the supplemental material (Figure S1 and Table S1). No modification of antimicrobial therapy due to toxicity or adverse events was required throughout the study.

## 4. Discussion

In this study, we analyzed the pharmacokinetics of meropenem, administered via two different infusion regimens in adults with CF experiencing a pulmonary exacerbation. The percentage of fT > 16 and fT > 32 mg/L was higher in the extended infusion group, with statistically significant prolongation of the fT > 32 mg/L (*p* = 0.037), whereas peak meropenem concentrations were significantly higher in the short infusion group. We preferred an infusion time of two hours rather than a 3-h or 4-h infusion as a comparator as this is more feasible to implement in a HIVAT schedule using an elastomeric infusion pump system (Easypump^®^, Braun, Melsungen, Germany) for self-application, according to our local standard.

Current international guidelines for Antimicrobial Stewardship recommend individualized dosing strategies and adaptation of doses according to measured serum levels as concepts to optimize anti-infective therapy in the critically ill [15,16]. Likewise, the recommendation to adjust the administration of beta-lactam antibiotics to prolonged or continuous infusion can be found increasingly more often in current literature [3,7]. The killing kinetic of carbapenems is time-dependent; therefore, free drug concentration time exceeding the fT > MIC is crucial [17]. The lower limit of meropenem level depends on the present pathogen and its resistance profile (target level 4–6 times the MIC) and, accordingly, differs individually [18].

The use of meropenem for treatment of pulmonary exacerbation has significantly contributed to improving the quality of life and life expectancy of people with CF [13]. It has been reported that half of people with CF infected by PA harbor multiple phenotypes of the microorganism [19]. A number of endemic strains have been described to be associated with lower susceptibility to antibiotics [20]. Thus, the antimicrobial efficacy of meropenem in treating MDR PA is of particular importance [5]. For example, in a MDR strain (MIC ≥ 8) the intended goal of meropenem levels would be 32–48 mg/L. A small study of 15 children with CF showed that the meropenem time above MIC exposure is predictive of response in pulmonary exacerbation resulting in improved post-treatment pulmonary function [21]. In addition, lower meropenem peak levels might reduce side effects, e.g., liver injury and neurotoxicity.

Our study has several limitations. First, we measured the plasmatic drug concentrations, which might be different from concentrations in the lungs as the site of infection. Second, our trial had a small sample size and a single-center design.

## 5. Conclusions

Our results demonstrate that in people with CF, longer fT > MIC can be achieved with a simple modification of meropenem dosing. Further randomized long-term studies are needed to clarify if this may translate into improved microbiological and clinical outcomes, in particular in adults with difficult-to-treat chronic infection by carbapenem-resistant *Pseudomonas aeruginosa*, and to prove its feasibility in HIVAT, applying commonly used elastomer pumps for self-application.

## Figures and Tables

**Table 1:** Key characteristics of people with cystic fibrosis (CF) in the two treatment groups.

	Short Infusion (30 min)	Extended Infusion (120 min)
	N = 6	N = 6
**Sex; *n* (%)**		
Female	2 (33)	3 (50)
Male	4 (67)	3 (50)
**Genotypes; *n* (%)**		
Homozygous F508del	2 (33)	2 (33)
Heterozygous F508del	2 (33)	3 (50)
Other	2 (33)	1 (17)
**Age; medium (SD)**	27.5 ± 8.04	26.67 ± 7.84
**BMI; medium (SD)**	20.07 ± 3.5	19.75 ± 2.71
**Best FEV1; medium (SD)**	36.67 ± 11.3	48 ± 15.67
**PA; *n* (%)**		
Yes	5 (83)	6 (100)
No	1 (17)	0 (0)
**3/4 MRGN PA; *n* (%)**	2 (33)/1 (17)	0 (0) / 3 (50)
**Combination therapy with tobramycin, *n* (%)**	5 (83)	6 (100)
**Laboratory results; medium (SD)**		
eGFR (mL/min)	129.83 ± 6.27	129 ± 12.39
ALT (U/L)	26.33 ± 18.91	39.33 ± 27.63
AST (U/L)	36.17 ± 30.22	25 ± 8.74
WBC (10^3^/µL)	11.55 ± 3.59	13.65 ± 3.15
CRP (mg/L)	32.81 ± 47.32	37.15 ± 40.21

Abbreviations; BMI: body mass index; FEV1: forced expiratory volume in 1 s; PA: *Pseudomonas aeruginosa*; 3/4 MRGN: 3/4 multidrug-resistant Gram-negative; eGFR: estimated glomerular filtration rate (creatinine-based; estimated by CKD-EPI-formula); ALT: alanine transaminase; AST: aspartate transaminase; CRP: C-reactive protein; WBC: white blood cells.

**Table 2:** Comparison of fT > MIC and peak concentrations between groups.

	Treatment Group		
	30 min	120 min	*p*-Value
Mean fT > 16 mg/L (min)	141 ± 49	200 ± 21	0.078
Mean fT > 32 mg/L (min)	82 ± 23	134 ± 43	0.037
C peak	107 ± 20	59 ± 20	0.005

Data are shown as mean ±SD. fT > MIC: time above the minimal inhibitory concentration; C peak: peak concentration of meropenem.

## Data Availability

The data presented in this study are available on request from the corresponding author. The data are not publicy due to data protection regulations.

## Supplementary Materials

The following are available online at https://www.mdpi.com/2079-6382/10/3/292/s1, Figure S1: Pharmacokinetics of meropenem-raw data, Table S1: Meropenem serum concentrations at predefined time points, Methods and Materials S1: Measuring systems, sample preparation and measurement, patient material.
