# Supplementary material for: Pharmacokinetics of Meropenem in People with Cystic Fibrosis—A Proof of Concept Clinical Trial
Source: Antibiotics (Basel). 2021 Mar 11;10(3):292. doi: 10.3390/antibiotics10030292 (PMC7998425; doi:10.3390/antibiotics10030292)
Supplement: Supplementary file 1 [file antibiotics-10-00292-s001.pdf]

Supplemental material

Figure S1: Pharmacokinetics of meropenem – raw data

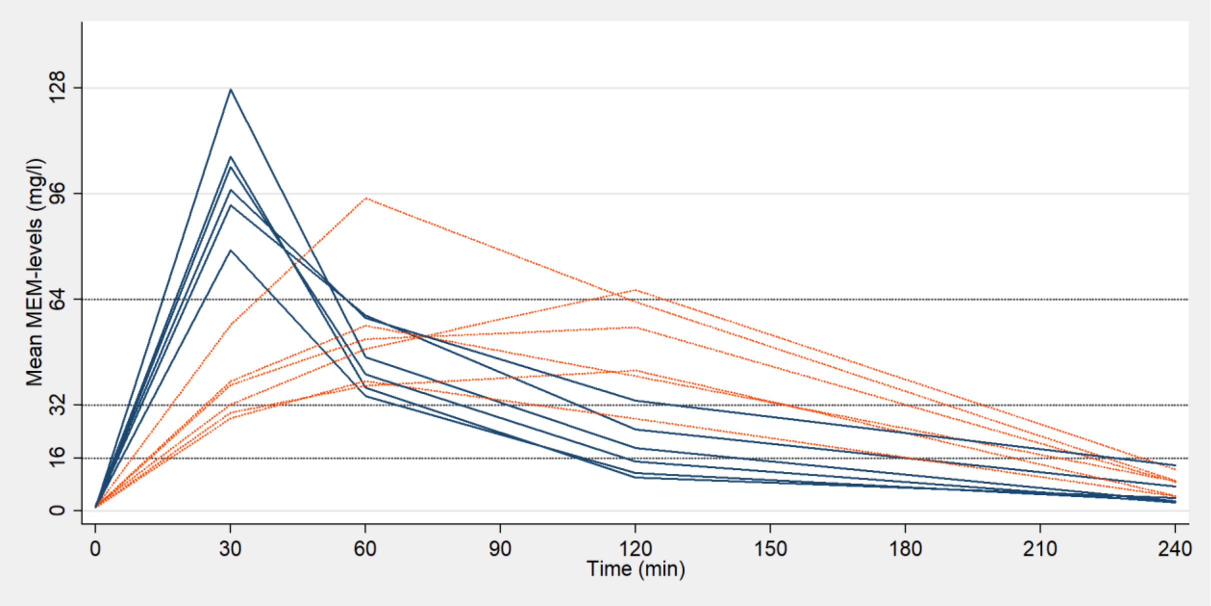

Pharmacokinetics are presented as “as is” per patient. Patients who received meropenem over 30/120 minutes are blue-/orange-colored, respectively.

Table S1: Meropenem serum concentrations at predefined time points

	0 min	30 min	60 min	120 min	240 min
patient #1 (30 min)	<1.1	127.6	46.6	19.1	2.9
patient #2 (30 min)	1.3	107.4	37.2	10.1	3.9
patient #3 (30 min)	<1.1	79.0	24.8	11.5	2.9
patient #4 (30 min)	<1.1	92.5	59.2	24.7	7.3
patient #5 (30 min)	<1.1	104.3	41.4	15	2.6
patient #6 (30 min)	1.6	97.2	58.4	33.3	13.8
patient #7 (120 min)	<1.1	56.3	94.7	63.3	9.1
patient #8 (120 min)	<1.1	29.7	37.9	42.5	4.5
patient #9 (120 min)	<1.1	28	39.3	27.9	4.4
patient #10 (120 min)	1.1	39.2	56.1	40.8	8.9
patient #11 (120 min)	1.5	32.2	49	66.9	12.5
patient #12 (120 min)	<1.1	38.1	52	55.6	8.7

Methods and Materials

Measuring systems

Analyses were performed on an Agilent 1200 series HPLC with UV detection at 290 nm after stabilisation of the sample material and subsequent protein precipitation (Agilent Technologies, Palo Alto, USA).

Sample preparation, reagents and measurement

Sample preparation and analysis were performed according to the IVD-CE certified kit Antibiotics in Serum/Plasma – HPLC (Chromsystems Instruments & Chemicals, Graefelfing, Germany). Measurement based on a 3+1 multiple point calibration. Two commercial quality controls were measured daily. Sample concentrations exceeding the measurement range were diluted according to the instruction sheet.

Patient material

Materials of investigation were heparinized plasma samples of patients. The samples were stored at --80 °C continuously until starting the analyses procedure.
